# Detection of ryanodine receptor G4911E and I4754M mutation sites and analysis of binding modes of diamide insecticides with RyR on *Galeruca daurica* (Coleoptera: Chrysomelidae)

**DOI:** 10.3389/fphys.2022.1107045

**Published:** 2022-12-22

**Authors:** Hao Ren, Hongling Zhang, Ruoyao Ni, Yanyan Li, Ling Li, Wenhe Wang, Yu Tian, Baoping Pang, Yao Tan

**Affiliations:** ^1^ Research Center for Grassland Entomology, Inner Mongolian Agricultural University, Hohhot, China; ^2^ State Key Laboratory of Integrated Management of Pest Insects and Rodents, Institute of Zoology, Chinese Academy of Sciences, Beijing, China; ^3^ Forestry station of Ar Horqin Banner, Chifeng, China; ^4^ Grassland Station of Xianghuang Banner, Xilinhot, China

**Keywords:** *Galeruca daurica*, ryanodine receptor, diamide insecticide, homologous modeling, molecular docking, binding modes

## Abstract

In recent years, the leaf beetle *Galeruca daurica* has broken out in the northern grasslands of Inner Mongolia, its management still mainly depends on chemical control using traditional insecticides or with novel action. The study was aim to identify mutation locus associated with resistance to diamide insecticides in field population of *G. daurica*, to provide a reference for rational selection of insecticides and to avoid the rapid resistance development to diamide insecticides. We cloned the full length of the ryanodine receptor gene of *G. daurica* (*GdRyR*), constructed 3D model and transmembrane regions by homologous modeling based on deduced amino acid sequence. Two potential mutation loci (Gly4911Glu and Ile4754Met) and allelic mutation frequencies were detected in individuals of *G. daurica*. In addition, their binding patterns to two diamide insecticides (chlorantraniliprole, cyantraniliprole) were analyzed separately using a molecular docking method. The full-length cDNA sequence of *GdRyR* (GenBank accession number: OP828593) was obtained by splicing and assembling, which is 15,399 bp in length and encodes 5,133 amino acids. The amino acid similarity of *GdRyR* with that of other Coleopteran insects were 86.70%–91.33%, which possessed the typical structural characteristics. An individual resistance allelic mutation frequency test on fifty field leaf beetles has identified 12% and 32% heterozygous individuals at two potential mutation loci Gly4911Glu and Ile4754Met, respectively. The affinity of the I4754M mutant model of *GdRyR* for chlorantraniliprole and cyantraniliprole was not significantly different from that of the wild type, and all had non-covalent interactions such as hydrogen bonding, hydrophobic interactions and π-cation interactions. However, the G4911E mutant model showed reduced affinity and reduced mode of action with two diamide insecticides, thus affecting the binding stability of the ryanodine receptor to the diamide insecticides. In conclusion, the G4911E mutation in *GdRyR* may be a potential mechanism for the development of resistance to diamide insecticides on *G. daurica* and should be a key concern for resistance risk assessment and reasonable applications of diamide insecticides for control in future. Moreover, this study could provide a reference for ryanodine receptor structure-based insecticides design.

## Introduction

*Galeruca daurica* (Coleoptera: Chrysomelidae), a serious grassland pest since its sudden outbreaks on the Inner Mongolia grasslands in 2009 (Zhou et al., 2016, Zhou et al., 2019), has continually spread and caused great losses to pasture on the Inner Mongolia grasslands (Ma et al., 2019, Ma et al., 2021), and levels of damage has increased year by year (Duan et al., 2022). The application of traditional chemical insecticides to control outbreaking pests in desert steppes has the advantages of a good insecticidal effect with rapid results, especially organophosphorus, pyrethroids, chloronicotinyl insecticide (Dong et al., 2016; Gao et al., 2022). Currently, the management of *G. daurica* is dependent on above-mentioned insecticides (Chang et al., 2015; Zhang et al., 2022). Besides, plant-derived insecticides have been reported to provide good protection against the key pest *G. daurica*, such as: neem, matrine and nicotine analogues (Chang et al., 2015; Du et al., 2016). In recent years, the chemical insecticides with novel action, high efficiency, high selectivity, low toxicity and low residue are being chosen to control this outbreaking insect in desert steppe. Among them, chlorantraniliprole has been reported to have strong insecticidal activity against the *G. daurica* and recommended for pest management (Zhang et al., 2022).

Diamide insecticides are a new class of highly effective, broad-spectrum, low toxicity, good systemic absorption and highly selective insecticides with O-amido benzamide chemical structure, mainly with stomach toxicity and high effectiveness against Coleoptera, Diptera and Lepidoptera insects (Tohnishi et al., 2005; Temple et al., 2009; Hughes et al., 2013; Huang et al., 2021). The main products currently on the market include: Chlorantraniliprole, Fluorobenzamide, Cyantraniliprole, Cyclaniliprole, Tetrachlorantraniliprole, Broflanilide, and Tetraniliprole (Sparks and Nauen, 2014). These chemical ligands can open calcium channels in insects by binding to the Ryanodine receptor (RyR), causing the freeing of large amounts of Ca^2+^ from the sarcoplasmic reticulum and endoplasmic reticulum, therefore, a significant reduction in the Ca^2+^ content triggers an imbalance in the insect’s internal environment, resulting in the cessation of feeding, functional muscle disorders, neurological disorders, and even death (Sattelle et al., 2008; Jeanguenat, 2013; Guo et al., 2021). RyR is a calcium channel protein distributed in the endoplasmic reticulum and sarcoplasmic reticulum of animal myocytes, and consists of a homotetrameric structure with four subunits containing 5,000 amino acid residues (Amador et al., 2009). Currently, diamide insecticides have been widely used against key agricultural insects, such as: diamond back moth *Plutella xylostella* (Guo et al., 2014, Guo et al., 2021), beet armyworm *Spodoptera exigua* (Zuo et al., 2017), *Spodoptera frugiperda* (Lv et al., 2021), rice stem borer Chilo suppressalis (Gao et al., 2013; Huang et al., 2020) and so on. Among them, chlorantraniliprole and flubendiamide were reported the most widely-used diamide insecticides with high insecticidal activity, low toxicity and safety to mammals and humans (Troczka et al., 2015).

In recent years, it has been continuously reported that the overuse of diamide insecticides led to the occurrence of resistance, with the resistance mechanism of the diamond back moth *P. xylostella* as a lepidopteran model pest being more intensively studied. Troczka et al. (2015) reported that the G4946E mutation in RyR was detected in resistant *P. xylostella* populations in Thailand and the Philippines, and was confirmed to affect the binding of diamide insecticides to RyR by *in vitro* expression in Sf9 cells. More recent studies have demonstrated that the I4790M/K mutation in PxRyR is associated with resistance to diamide insecticides in field populations of diamond back moth (Guo et al., 2014; Jouraku et al., 2020; Wang et al., 2020; Jiang et al., 2021; Richardson et al., 2021). It was reported that the high frequencies of mutations were found among six Chinese field populations collected from 2016 to 2019 resulting in RyR I4743M substitutions on *Spodoptera exigua*, however, no significant correlation was found between chlorantraniliprole resistance level and SeRyR I4743M allele frequency but CYP9A186 F116V substitutions (Teng et al., 2022). Boaventura et al. (2019) reported that a I4734M mutation of the FAW ryanodine receptor (RyR) C-terminal domains II to VI has recently been described to confer target-site resistance to diamides in Lepidopteran insect *Spodoptera frugiperda*. Among the reported mutant loci of the insect ryanodine receptor involving resistance to diamide insecticides, G4946E and I4790M on *P. xylostella* are the classical allele substitutions with the highest frequency of resistance, corresponding to G4911 and I4754 of RyR in *G. daurica*, respectively. It has not been reported that resistance occurrence, resistance levels of diamide insecticides on *G. daurica*, whether there are amino acid changes in *GdRyR* in field populations and whether they may contribute to resistance to diamide insecticides.

With the development of bioinformatics and computer simulation technology, protein homology modeling, molecular docking and other technical tools are becoming increasingly sophisticated. Novel ideas for studying pest resistance mechanisms to insecticides are proposed by predicting the binding pattern of receptors and ligands (Chen et al., 2021). Homology modeling uses the crystal structure of a protein molecule with high homology to its amino acid sequence as a template for sequence alignment, and predicts the structure of an unknown, homologous protein from the conserved domain of known crystal protein molecule (Boris and Ke, 2022). At present, the crystal structure of the *GdRyR* protein has not been identified, but the structure analysis of some mammalian ryanodine receptors is more mature and can be used as a reference approach for structural simulation and molecular docking (Ma et al., 2020). The deduced amino acid sequence was obtained from the cloned *GdRyR* gene sequence, and homology modeling and molecular docking were applied to construct the 3D model and analyze its affinity conformation, respectively.

In the study, we cloned the full-length *GdRyR* gene, examined the two classic mutation allele frequency of *GdRyR* associated with resistance to diamide insecticides in individuals of field populations of *G. daurica*, and applied homology modeling to construct a three-dimensional model of *GdRyR* and its transmembrane region. The binding patterns of *GdRyR* and diamide insecticides were studied by molecular docking, and the relationship between potential mutation sites of *GdRyR* and resistance was analyzed. The aim of this study was to explore the mechanism of resistance to diamide insecticides from the perspective of structural biology, and to lay a theoretical foundation for further analysis of the resistance mechanism of diamide insecticides.

## Materials and methods

### Insect rearing and sample preparation

The overwintering eggs of *G. daurica* were collected from the Xianghuang Banner grasslands (44°62′N, 115°80′E) of Inner Mongolia, China on September 2021, were brought back to the Research Center for Grassland Entomology (40°48′N, 111°42′E), Inner Mongolia Agricultural University (Hohhot, Inner Mongolia, China), and were maintained in intelligent light artificial incubator (PRX-350C, Ningbo HaiShuSaiFu experimental instrument Co., Ltd.) (25°C ± 1°C, RH = 70 ± 10%, L14:D10). After hatching out, the larvae were fed on *Allium mongolicum* in a incubator under the same conditions just as described by Tan et al. (2018). The healthy third instar larvae in consistent physiological state were collected for further experiments.

### Bioassay of chlorantraniliprole

Bioassays were conducted using a leaf-dip method based on methods of He et al. (2012) and Zhang et al. (2022) with minor modifications. 95% chlorantraniliprole (DuPont) was dissolved in acetone solution, and then diluted into five to six concentrations with distilled water containing .05% Triton X-100 at a triple gradient dose. The .05% Triton X-100 solution was used as blank control. The fresh leek cultured in the laboratory measuring 5 cm × .5 cm were immersed in the prepared various concentrations of chlorantraniliprole for 15 s, and taken out in ventilated cupboard to air dry and then placed into a Petri dish lined with filter paper. Twenty healthy 2-day third instar larvae were placed into each Petri dish with six pieces of leek leaves with four replications for toxicity assessment bioassays. Mortality was assessed after 48 h of exposure, larvae that did not move when gently pushed with a fine hair brush were considered dead. The control groups was below 5%, mortality was corrected using Abbott’s formula (Abbott, 1925). The LC_50_ value and slope were calculated by regression probit analysis conducted with the POLO-Plus software (2002).

### Cloning of *GdRyR* gene

Sequences annotated as ryanodine receptor genes were screened from the transcriptome data published in Genbank (Bioproject No. 785282), and specific primers listed in Table 1 were designed for segmental cloning. The Total RNA was extracted from the whole body using RNAiso reagent (TaKaRa, Dalian, China) based on the manufacturer’s instructions, cDNA was synthesized by reverse transcription using the PrimeScript^®^ RT Reagent Kit with gDNA Eraser (TaKaRa, Dalian, China) and as the PCR templates. Total PCR reaction system (50 μl) included cDNA template (2 μl), forward and reverse primers (1 μl, 0.2 μmol/L), amplification enzyme mix (25 μl), RNAse Free ddH_2_O (21 μl). 2 × Vazyme LAmp^®^ Master Mix was purchased online from Vazyme Co., Ltd. and used for high fidelity and rapid PCR, the annealing velocity is high to 1 kb/s. PCR amplification conditions were performed as follows: 98°C for 3 min, followed by 35 cycles of 98°C for 10 s, 60°C for X s (X: each primer used a different annealing time), and 72°C for 3 min. Finally, it was extended for 5–10 min at 72°C (Table 1). The expected-size fragments were purified, ligated into pMD19-T vector (TaKaRa, code: D102A), and positive transformants were selected for plasmid isolation using MiniBEST Plasmid Purification Kit (TaKaRa, Dalian, China) and sent to Sangon Biotech company for sequencing. The fragments were overlapped and aligned with the annotated *GdRyR* gene identified from the transcriptome database using DNAMAN software, and the spliced complete CDS sequence of the *GdRyR* gene was submitted to the NCBI database, the Genbank accession number is OP828593.

**TABLE 1:** Primers used in the study.

Primer name	Primer sequences (5′-3′)	Target fragment size (bp)	PCR working procedure	Purpose
RyR1-F	TAC​AAA​AAA​AAA​ACC​TAA​ACC​TCC	4,892	98°C, 3°min; (98°C, 10 s	Amplification of *RyR* gene fragment
RyR1-R	CTG​TCA​ACG​CCA​TCA​ATA​ACC​CTA			
			60°C, 30°s; 72°C, 3 min)*35	
			72°C, 10 min	
RyR2-F	AAT​CCT​ACT​GCT​ACT​CAA​CCA​CTC	5,664	98°C, 3°min; (98°C, 10 s	
RyR2-R	GCT​GTA​AGT​CTT​CTC​GCT​ATC​AAC			
			60°C, 45°s; 72°C, 3 min)*35	
			72°C, 10 min	
RyR3-F	GTC​TTT​GTT​TTA​TGT​GAA​TAC​CGT	4,325	98°C, 3°min; (98°C, 10 s	
RyR3-R	GGA​ATG​CCT​TTG​CTA​CCA​CTG​CCG			
			60°C, 30°s; 72°C, 3 min)*35	
			72°C, 10 min	
RyR4-F	CAT​AGG​GCA​GTG​TCA​TTC​CTC​GCT	1,544	98°C, 3°min; (98°C, 10 s	
RyR4-R	TTG​CTG​TTG​GGG​GCT​CGT​AAA​GTT			
			60°C, 20°s; 72°C, 1 min)*35	
			72°C, 5 min	
S1-F	CAT​AGG​GCA​GTG​TCA​TTC​CTC​GCT	234	98°C, 3°min; (98°C, 10 s	Mutation site frequency detecting
S1-R	TAA​ATC​GTC​ATC​TTC​TGG​CTG​TTC			
S2-F	AGA​TAA​TGC​TTT​CCT​GTA​TTC​CTT	250		
			60°C, 15°s; 72°C, 20°s)*30	
S2-R	GTC​TTC​TTC​TTG​AAC​ATA​AAA​CTT			
			72°C,5 min	

### Bioinformatics analysis of *GdRyR* gene

ORF Finder was applied to search the open reading frame of the *GdRyR* gene, and sequence integrity was verified by NCBI’s Blastx tool to predict the gene isoelectric point, molecular mass, atomic composition, protein structural domain, signal peptide, and transmembrane region. Phylogenetic tree was constructed using the BLAST tool and DNAMAN V6.0 software to align amino acid sequence homology, using the neighbor-joining method in MEGA software and repeatedly running 1,000 times.

### Detection the G4911E and I4754M Allelic Mutation Frequencies in the Field Population

The total RNA was isolated from individuals of *G. daurica* population collected from the Xianghuang Banner grasslands for cDNA preparation and PCR. The two pairs of specific primers containing the potential mutation sites G4911 and I4754 were designed based on the sequence of the C-terminal transmembrane region (Table 1). The specific bands obtained by PCR amplification were purified and sent to company for sequencing, and the sequences from different individuals were compared in the point mutation region to detect the frequency of allelic mutation sites.

### Homologous modeling of *GdRyR* and molecular docking

#### Homologous modeling and model evaluation

The *GdRyR* amino acid sequence was submitted to the SWISS-MODEL server, and the predicted rabbit RyR cryo-electron microscopy structure (PDB: 7CF9) with 61.8% amino acid sequence homology to *GdRyR* reported by Ma et al. (2020) was used as a template to construct a model of *GdRyR* using Modeller 10.2. The Align2D module was used to compare *GdRyR* with the template sequence, and then the Model-single module was used to build 100 candidate models. The stability of the models was measured by the Dope parameter, and the model with the lowest energy was selected as the optimal model for homology modeling, which was named as WT *GdRyR*. To obtain more accurate 3D structure for molecular docking, the amino acid sequence of the C-terminus of *GdRyR* was submitted on the AlphaFold2 platform on the Wemol online website, and the AlphaFold2 tool was used to construct a protein model of the C-terminus of *GdRyR* (WT *GdRyR*-C). The 3D structures of protein crystals obtained from Modeller and AlphaFold2 simulations were verified for stereochemical plausibility and energetic stability using the Molprobity tool. The rationality of the *Pis* and *Phi* angles between residues was demonstrated by the Ramachandran Plot, and the rationality of the protein model was evaluated based on the percentage of optimal regions, generally allowed regions, *etc.* in the Ramachandran Plot (Williams et al., 2018).

### Construction of *GdRyR* mutants

The amino acid sequence of *GdRyR* was compared with domains containing mutation loci for *P. xylostella* RyR (G4946E, I4790M), *S. frugiperda* for (G4900E, I4743M) and *S. exigua* (G4891E, I4743M) (Zou et al., 2019), which correspond to G4900E, I4754M mutation sites of *GdRyR*. Mutation of “Ile” was replaced by “Met” in I4754 in *GdRyR* using AlphaFold2, the mutation model *GdRyR*-C-M4754 was constructed. Similarly, “Gly” was substituted into “Glu” in G4900 in *GdRyR*, and the mutation model *GdRyR*-C-E4900 was constructed. The models WT *GdRyR*-C, *GdRyR*-C-M4754 and *GdRyR*-C-E4911were processed separately using Autodock Tool-1.5.7 for energy minimization and dehydrogenation as molecular docking receptors.

### Ligand Construction

The 3D structures of chlorantraniliprole and cyanobacteriamide were downloaded from the PubChem website, and converted into a suitable format for molecular docking using Open Babel-2.4.1, and used as ligands for molecular docking using Autodock Tool-1.5.6 for energy minimization *etc.*

### Molecular docking and binding mModel analysis

Based on the reported binding sites of the rabbit RyR complex crystal structure with chlorantraniliprole, the active pockets of the constructed 3D model were predicted and the docking-box was constructed. The box parameters used for the docking of *GdRyR*s were as follows: center-x = 8.18, center-y = −1.56, center-z = .877; size-x = 99.75, size-y = 99.75, size-z = 99.75. The processed protein receptors were docked to the ligands using Autodock vina1.1.2 software. Computing platform was as follows: Microsoft-PC: Intel(R) Core(TM) i5-1035G4CPU@1.10GHz 1.50 GHz.

By molecular docking, the two ligands were selected for optimal conformation to construct complexes with the ryanodine receptor, and the binding modes were analyzed using the PLIP online tool, including: hydrogen bonding, hydrophobic interactions, π-π, π-cation, halogen bonding and non-covalent interaction forces such as interactions. Pymol-2.1.0 was used to visualize the binding modes.

### Computing software and online tools

Homologous sequence alignment: https://blast.ncbi.nlm.nih.gov/Blast.cgi; Predicting protein properties such as gene isoelectric point, molecular mass and atomic composition: ProtParam online tool; Protein domain prediction: https://www.ebi.ac.uk/interpro/result/InterProScan/(InterProscan); Signal peptide prediction: http://www.cbs.dtu.dk/services/SigRyRlP/; Homologous modeling: https://swissmodel.expasy.org/(SWISS-MODEL) (Waterhouse et al., 2018); Protein quality evaluation: https://Main page-MolProbity (duke.edu) (Molprobity) (Williams et al., 2018); Protein crystal structure PDB data: http://www1.rcsb.org/; NCBI databse: https://www.ncbi.nlm.nih.gov/; Transmembrane region prediction (TMHMM): http://www.cbs.dtu.dk/services/TMHMM/(Möller et al., 2001); Action force analysis tools (PLIP): https://projects.biotec.tu-dresden.de/plip-web/plip (Melissa et al., 2021); Small molecular crystal structure: PubChem (nih.gov ).

MEGA X was applied to construct phylogenetic tree (Kumar et al., 2018); Open Babel-2.4.1 was used to convert the file format of the chemical structure type (O’Boyle et al., 2011); The 3D protein crystal structure was visualized by Pymol-2.1.0 (Delano, 2002); Modeller (Version-10.2) was used for homologous modeling (Chen et al., 2021); Autodock Tool-1.5.7 (Morris et al., 2009) and Autodock vina 1.1.2 (Trott and Olson., 2010) were used for molecular docking; AlphaFold2 was applied for deep learning modeling (Jumper et al., 2021).

## Results

### Toxicity of chlorantraniliprole

Linear regression of the dose–mortality relationship (Y = −2.557 ± 2.291 X) was fitted to the observed data (i.e. no significant deviation between the observed and the expected data; *χ* ^*2*^ = 1.81, d*f* = 3) and LC_50_ was considered valid (inf *lim* < LC_50_ < sup *lim*: 21.25 < 44.10 mg/L < 80.33).

### Gene cloning and sequence analysis of *GdRyR* gene

**FIGURE 1:**
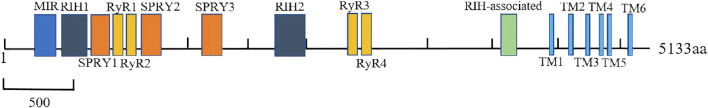
The schematic diagrams of conserved gene domains of *GdRyR*.

Based on the *GdRyR* sequence information in the transcriptome, a total of four target fragments were amplified. After overlapped, a complete open reading frame (ORF) of 15,399 bp, encoding 5,133 amino acids, was obtained by BlastX alignment and sequence splicing. The molecular weight of the protein is 582.53 kDa, and the isoelectric point (pI) is 5.51. The *GdRyR* consists of five elements: carbon (C), hydrogen (H), Nitrogen (N), Oxygen (O) and Sulphur (S), and the total number of atoms is 81,490, the chemical formula is C_258925_H_40538_N_6992_O_7799_S_236,_ it has six transmembrane structure regions and no signal peptide. As shown in Figure 1, the N-terminal part of the gene contains one MIR domain (Mannosyltransferase, IP3R and RyR) located between amino acid sites 220-397, two RIH domains (RyR and IP3R Homology) located at amino acid sites 447-642 and 2254-2484, respectively, and three SPRY domains (SPla and RyR) located at amino acid sites 664-802, 1091-1214 and 1563-1706, respectively. The gene also has four RyR domains located at amino acid sites 854-944, 967-1056, 2861-2952, 2979-3062, and one highly conserved RIH-associated domain located at amino acid sites 4021–4137 before the transmembrane helix; The C-terminus has six transmembrane helices (TM1 to TM6) located at amino acid sites 4474-4496, 4658-4680, 4739-4761, 4881-4903, 4929-4951, and 5009-5028, respectively.

### Sequence homology analysis of *GdRyR* gene

**FIGURE 2:**
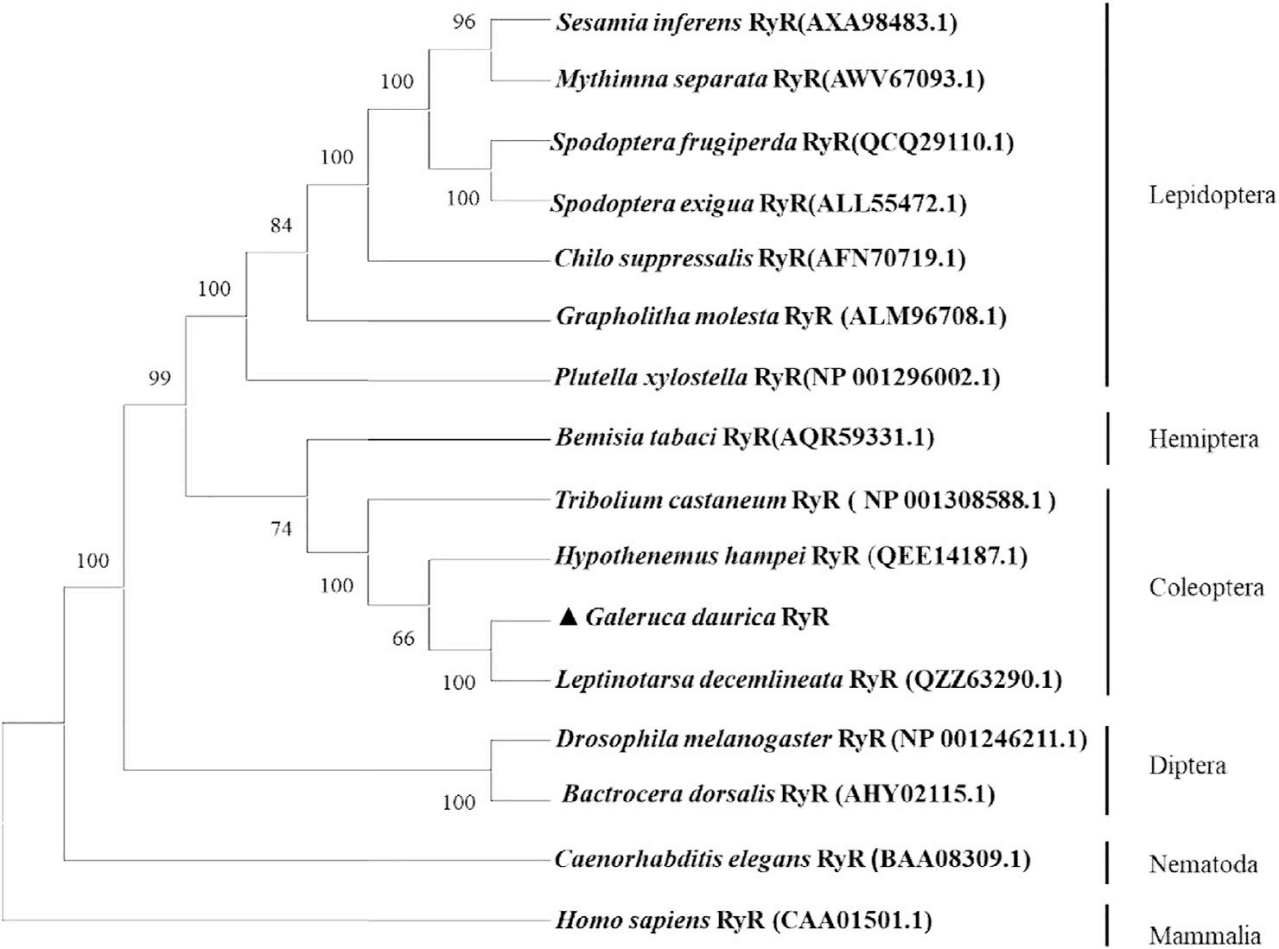
Phylogenetic tree of ryanodine receptor amino acid sequences from *G. daurica* and other insects. Note: The tree was generated by MEGA X using the Neighbor-Joning (NJ) method, using model p-distance. The topology was tested using bootstrap analyses (1,000 replicates). Numbers at nodes are bootstrap values.

The amino acid sequences encoded by the *GdRyR* gene were blasted with RyR of other insect species researched in the NCBI database, and a phylogenetic tree was constructed using the neighbour-joining (NJ) method. Figure 2 showed that *GdRyR* has the highest homology with RyR of potato beetle *Leptinotarsa decemlineata* (GenBank Accession No: QZZ63290.1), and amino acid sequence identity is 91.33%. The amino acid homology with *Hypothenemus hampei* RyR (QEE14187.1) and *Tribolium castaneum* RyR (NP 001308588.1) were 88.05% and 86.70%, respectively, and the RyR of the four Coleopteran species clustered as one clade. The clustering results indicated that the *GdRyR* gene is highly similar to taxonomically similar insect taxa.

### Analysis of the G4911E and I4754M allelic mutation frequencies

The two potential allelic mutation sites and the occurrence frequency of Gly4911Glu and Ile4754Met were examined by individual extraction of RNA, using reverse-transcribed cDNA as a template, respectively. Fifty beetles collected from the grasslands of Xilingol Xianghuang Banner, Inner Mongolia, were conducted to gene sequence testing, the mutation loci and allelic frequencies of the *GdRyR* are summarized in Table 2. The results showed that only the heterozygous genotype G4911E was present in the test population, accounting for approximately 12% of the population; the heterozygous and homozygous genotypes of I4754M were present in 32% and 2% of the population, respectively.

**TABLE 2:** Allelic frequencies of point mutations on *GdRyR* from field population.

	G4911E				I4754M		
Genotype	GGT, 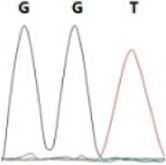	GGT/A, 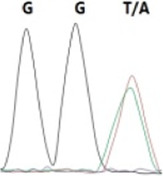	GGA, 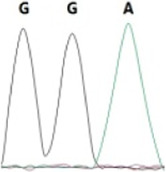	GG/AA, 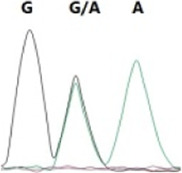	ATA, 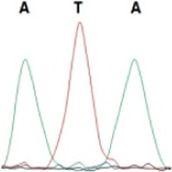	ATA/G, 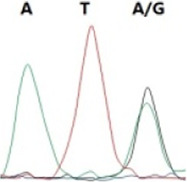	ATG, 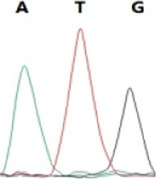
Amino acid	Gly(G)	Gly(G)	Gly(G)	Gly/Glu (G/E)	Ile(I)	Ile/Met (I/M)	Met(M)
Number of test leaf beetles	22	13	9	6	33	16	1
Point mutation frequency	44%	26%	18%	12%	66%	32%	2%

### Analysis and evaluation of homologous modeling

**FIGURE 3:**
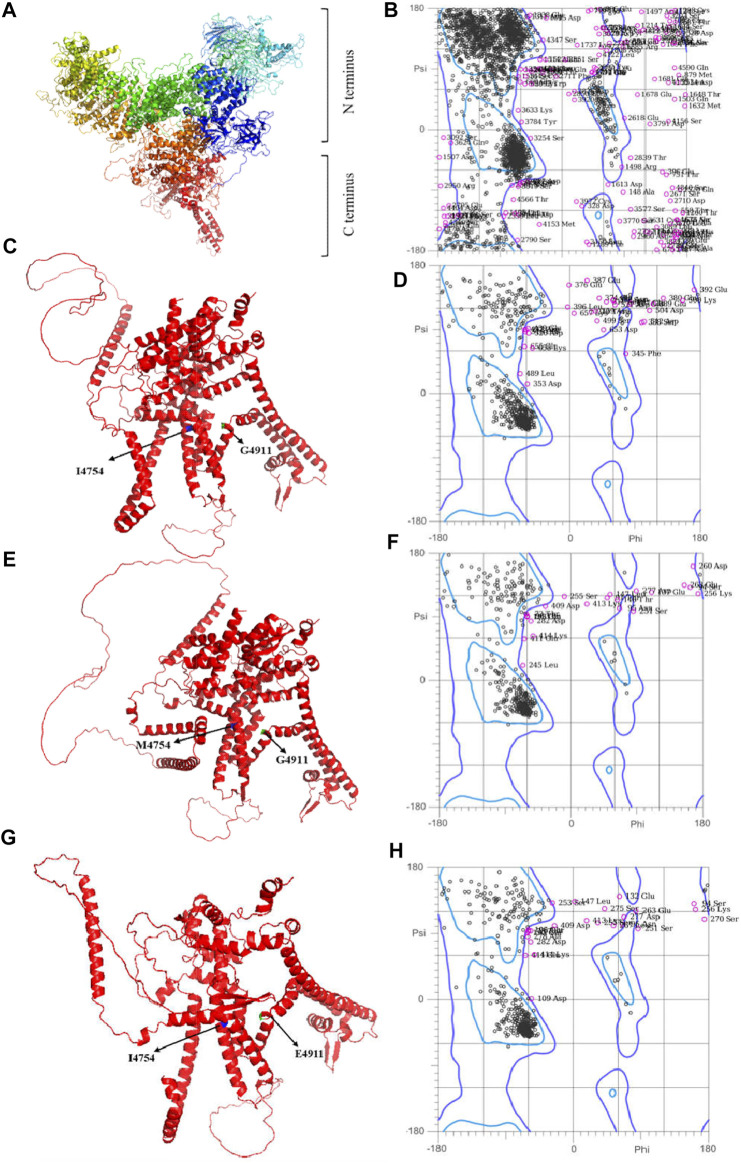
The models and evaluation results of WT *GdRyR*, WT *GdRyR*-C, *GdRyR*-C-M4754, and *GdRyR*-C-E4911. Note: **(A)** Three-dimension RyR model of WT *GdRyR*; **(B)** The result of Molprobity evaluation of WT *GdRyR*; **(C)** Three-dimension RyR model of the C-terminal transmembrane region of WT *GdRyR*-C; **(D)** The result of Molprobity evaluation of WT *GdRyR*-C; **(E)** Three-dimension RyR model of *GdRyR*-C-M4954; **(F)** The results of Molprobity evaluation of *GdRyR*-C-M4954. **(G)** Three-dimension RyR model of *GdRyR*-C-E4911; **(H)** The results of Molprobity evaluation of *GdRyR*-C-E4911.

The Modeller and AlphaFold2 are applied to construct the *GdRyR* 3D crystal structure (WT *GdRyR*), the C-terminal wild-type 3D structure of *GdRyR* (WT *GdRyR*-C) and the mutant structure (*GdRyR*-C-M4754) (*GdRyR*-C-E4911), respectively. Figure 3A showed the 3D structure of *GdRyR* consists of multiple α-helices, β-folds, β-turns, irregular curls, and some extended extension structures. In lateral view, the protein crystal structure resembles the letter “Y,” with a wide N-terminus and a narrow C-terminus, separated by a small angle at the C-terminus. As it can be seen from Figures 3C, E, G that the 3D structure of the C-terminal region of *GdRyR* is mainly composed of several parallel sets of α-helices, exhibiting obvious transmembrane protein properties. The 3D structural model of Ramachandran Plot diagram and the test results are shown in Figure 3 and Table 3, respectively. The percentages of residues in the optimal regions for WT *GdRyR*, WT *GdRyR*-C and *GdRyR*-C-M4754 and *GdRyR*-C-E4911 were 86.9%, 88.3%, 87.8%, and 88.1%, respectively. The proportions of amino acid residues in the acceptable region were 95.8%, 94.6%, 94.9%, and 94.6% respectively, all are greater than 90%, indicating that the constructed models were all reasonable.

**TABLE 3:** The results of Molprobity evaluation of *GdRyR*.

3D model	Favored regions (98 (%)	Allowed regions (>99.8 (%)
WT *GdRyR*	86.9	95.8
WT *GdRyR*-C	88.3	94.6
*GdRyR*-C-M4754	87.8	94.9
*GdRyR*-C-E4911	88.1	94.6

### Analysis of binding modes of diamide insecticides with *GdRyR*

**FIGURE 4:**
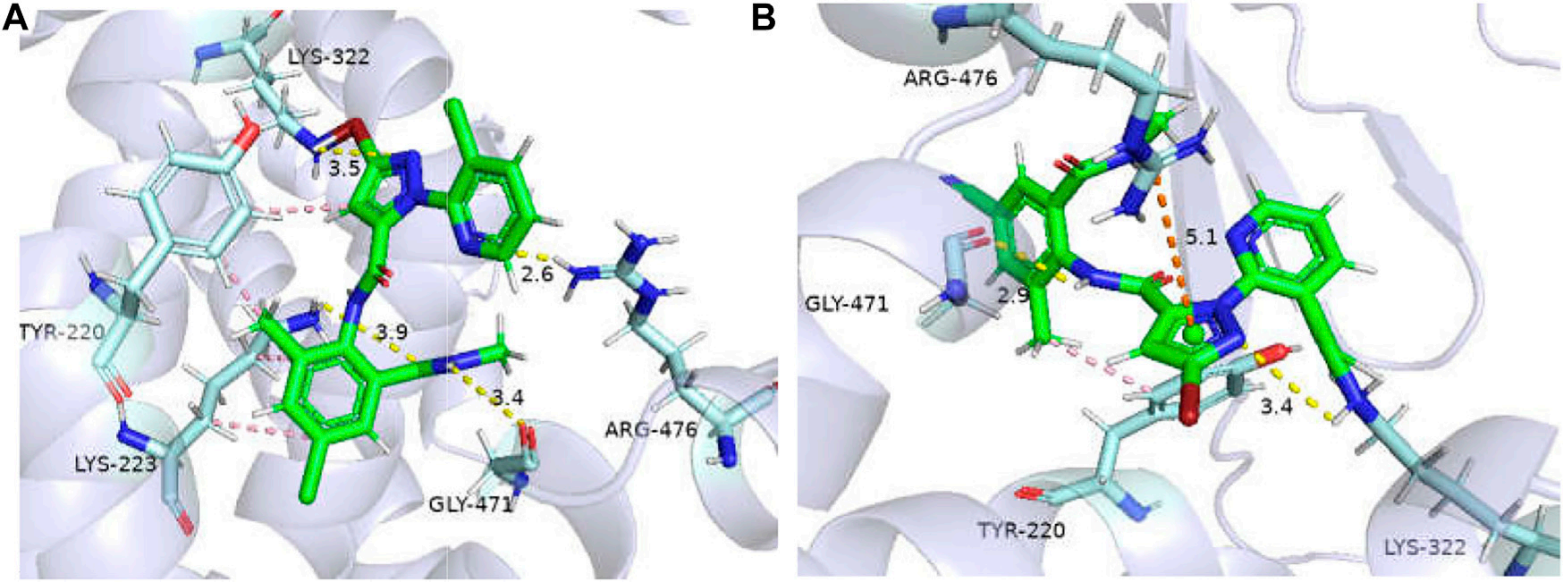
Binding modes of WT *GdRyR* and chlorantraniliprole **(A)** and cyanobacteriamide **(B)**. Note: Hydrophobic effect: pink; Hydrogen bond: yellow; π-cation: orange; Hydrogen: white; Oxygen: red; Nitrogen: blue; Selphur: dark orange.

The binding affinities of WT *GdRyR*-C to the optimal conformation of chlorantraniliprole and cyantraniliprole were −31.38, −33. 89 kJ/mol, respectively. The binding pattern was analyzed using PLIP and drawn by Pymol. As Figure 4 shows: Y4660 (Tyr-220), K4663 (Lys-223) form a hydrophobic interaction with the benzene and pyrazole rings of chlorantraniliprole, K4663 forms a hydrogen bond with the oxygen atom on the carbonyl group, K4762 (Lys-322) forms a hydrogen bond with the nitrogen atom of the pyrazole ring, G4911 (Gly-471) forms a hydrogen bond with the NH of the methylamino structure on the benzene ring, R4916 (Arg-476) forms a hydrogen bond with the nitrogen atom of the pyridine ring (Figure 4A). Y4660 forms a hydrophobic interaction with the methyl group on the benzene ring of cyantraniliprole, K4762 forms a hydrogen bond with the nitrogen atom of the pyrazole ring, G4911 forms a hydrogen bond with the NH of the amide structure on the side of the pyrazole ring, the nitrogen atom of R4916 forms a π-cation with the pyrazole ring of cyantraniliprole (Figure 4B).

**FIGURE 5:**
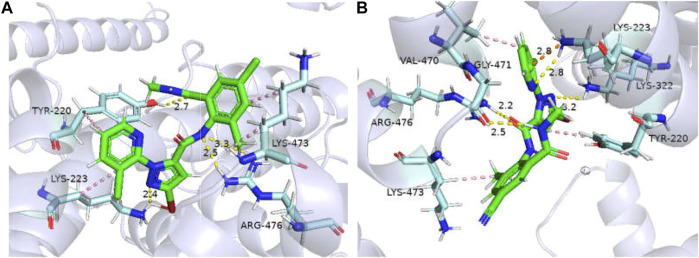
Binding modes of *GdRyR*-M4754 and chlorantraniliprole **(A)** and cyanobacteriamide **(B)**. Note: Hydrophobic effect: pink; Hydrogen bond: yellow; π-cation: orange; Hydrogen: white; Oxygen: red; Nitrogen: blue; Selphur: dark orange.

The binding affinities of *GdRyR*-M4754 to the optimal conformation of chlorantraniliprole and cyantraniliprole were −32.23, −33. 91 kJ/mol, respectively. Combined pattern analysis diagram was seen in Figure 5: Y4660 (Tyr-220) and K4663 (Lys-223) form hydrophobic interactions with the pyridine ring of chlorantraniliprole, K4913 (Lys-473) forms hydrophobic interactions with the benzene ring as well as the methyl group, Y4660 forms a hydrogen bond with the oxygen atom on the carbonyl group of the benzene ring, K4663 forms a hydrogen bond with the nitrogen atom of the pyrazole ring, R4916 (Arg-476) forms a hydrogen bond with the NH of the amide structure on the pyrazole side (Figure 5A). Y4660, V4910 (Val-470) and K4663 form hydrophobic interactions with the pyridine ring, pyrazole ring and methyl group of cyantraniliprole, respectively. K4663 forms a hydrogen bond with nitrogen on the pyridine ring, K4762 (Lys-332) forms a hydrogen bond with the nitrogen atom of the pyrazole ring, G4911 (Gly-471) forms a hydrogen bond with NH of the amide structure on the benzene ring side, R4916 forms a hydrogen bond with the oxygen atom of the carbonyl group on the pyrazole ring side, the nitrogen atom of K4663 forms a π-cation interaction with the pyridine ring of cyantraniliprole (Figure 5B).

**FIGURE 6:**
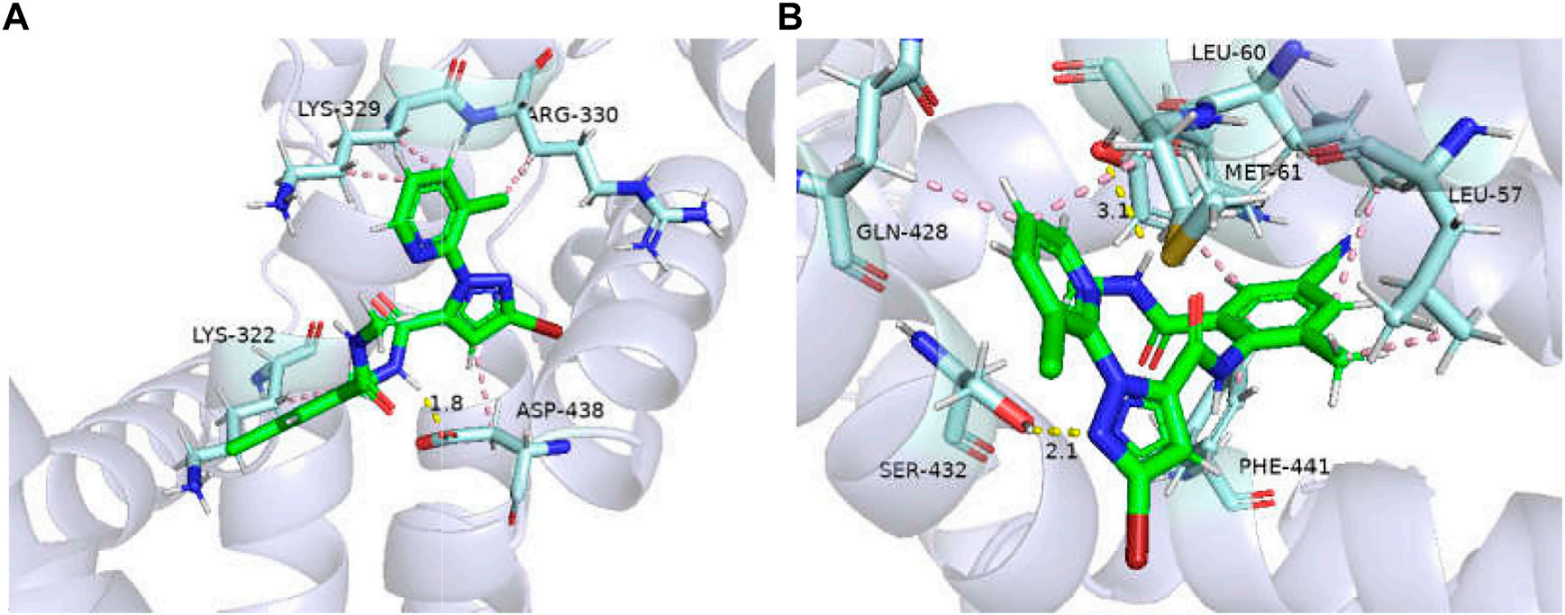
Binding modes of *GdRyR*-E4911 and chlorantraniliprole **(A)** and cyanobacteriamide **(B)**. Note: Hydrophobic effect: pink; Hydrogen bond: yellow; π-cation: orange; Hydrogen: white; Oxygen: red; Nitrogen: blue; Selphur: dark orange.

The binding affinities of *GdRyR*-E4911 to the optimal conformation of chlorantraniliprole and cyantraniliprole were −25.89, −27.54 kJ/mol, respectively. Combined pattern analysis diagram was seen in Figure 6: K4762 (Lys-329) forms hydrophobic interactions with methyl of chlorantraniliprole, K4769 (Lys-322) and R4770 (Arg-330) form hydrophobic interactions with the pyridine ring and the chlorine ion on the pyridine ring, respectively. D4878 (Asp-438) forms hydrophobic interactions and hydrogen bonds with the pyrazole ring and the NH of the pyrazole side amide structure, respectively (Figure 6A). L4497 (Leu-60) and L4500 (Leu-57) form hydrophobic interactions with the methyl and benzene rings of cyantraniliprole, respectively. M4501 (Met-61) and Q4868 (Gln-428) form hydrophobic interactions with the pyridine ring, Y4759 (Tyr-341) and F4881 (Phe-441) form hydrophobic interactions with the benzene ring, Y4759 forms a hydrogen bond with the NH of the amide structure on the side of the benzene ring, Q4868 (Gln-428) forms a hydrogen bond with the nitrogen atom of the pyrazole ring (Figure 6B).

### Analysis of mutation sites of *GdRyR*

As shown in Table 4: The mutation of isoleucine (Ile) to methionine (Met) at position 4754 in *GdRyR* showed no decrease in binding affinity to diamide insecticides; the mutation of glycine (Gly) to glutamic acid (Glu) at position 4911 in *GdRyR* showed a significant decrease in binding affinity to diamide insecticides. The binding modes of two diamide insecticides with WT *GdRyR*, *GDRYR*-M4754 and *GDRYR*-E4911 are shown in Figures 4–6: After mutating isoleucine (Ile, position 4754) of *GdRyR* to methionine (Met), the mode of interaction with diamide insecticides, the residues involved in the formation of the action force and WT *GdRyR* are essentially the same, mainly because the isoleucine (Ile) at position 4754 of WT *GdRyR* is not involved in the formation of the force action between ligands and receptors. The mutation to glutamic acid (Glu) in WT *GdRyR*, which has a hydrogen bonding interaction between glycine (Gly) at position 4911 and the diamide insecticide, prevents the formation of hydrogen bonds with the diamide insecticide, resulting in a reduced receptor-ligand interaction and decreased affinity. The mutations of amino acid from Gly to Glu at position 4911 in WT *GdRyR* may lead to resistance to diamide insecticides on *G. daurica*, while further studies are needed to determine whether the I4734M mutation is associated with resistance.

**TABLE 4:** The binding affinity changes of the wild-type (WT) and mutant *GdRyR*s and diamides.

Diamide insecticide	Binding affinity ±SE/(kJ/mol)		
	WT *GdRyR*	*GdRyR*-M4754	*GdRyR*-E4911
Chlorantraniliprole	−31.38 ± 0.14a	−32.32 ± 0.24a	−25.89 ± 0.29 b
Cyantraniliprole	−33.89 ± 032a	−33.91 ± 0.46a	−27.54 ± 0.32 b

Each value represents the mean (±SE) of ten replicates. The values (Mean ± SE) followed by different letters in the same column are significantly different between mutation at 5% significance level using ANOVA, followed by Dunnett T3 correction.

## Disscussion

The insect ryanodine receptor, one of the largest Ca^2+^ releasing channels, has an important role in muscle excitation-contraction coupling and is an important action target of diamide insecticides. Currently, in addition to the model insect *Drosophila*, the RyR genes of *P. xylostella* (Sun et al., 2012), *Cnaphalocrocis medinalis* (Wang et al., 2012), *Ostrinia furnacalis* (Cui et al., 2013), *S. exigua* (Zuo et al., 2017), *C. suppressalis* (Gao et al., 2013; Huang et al., 2021) and *S. frugiperda* (Li et al., 2021) have been successfully cloned, and different mutant sites have been reported to be associated with diamide insecticide resistance. In this study, the full-length cDNA sequence of the *GdRyR* gene was obtained by segmental cloning technique, which was assembled by splicing. Sequence homology analysis showed that *GdRyR* had 86.70%, 88.05%, and 91.33% amino acid similarity with the ryanodine receptors of the other Coleopteran insects, *T*. *castaneum* (Herbst), *H*. *hampei* (Ferrari) and *L*. *decemlineata* (Say), respectively. The structural domains were predicted to have one MIR structural domain, two RIH structural domains, three SPRY structural domains, four RyR structural domains and one RIH-associated structural domain at the N-terminal part of *GdRyR*, and six hydrophobic transmembrane motifs at the C-terminal end of *GdRyR*, this result is highly similar to the reported positions of the functional structural domains of insects such as *Carposina sasakii* (Sun et al., 2012), *C*. *suppressalis* (Peng et al., 2017). The MIR structural domain functions as a transferring ligand in mammals, and the RIH and MIR structural domains are involved in the formation of IP3 binding sites in IP3Rs (Ponting, 2000). The SPRY structural domain is often considered to have a function in regulating protein interactions (Woo et al., 2006; Cui et al., 2009). These structural features are similar to those of *Drosophila* DmRyR, suggesting that the *GdRyR* gene can encode a functional Ca^2+^ channel protein (Xu et al., 2000; Bouakaz et al., 2002).

Diamide insecticides, a class of chemical insecticides acting on insect ryanodine receptors, have good efficacy against a wide range of pests and are widely used in the chemical control on Lepidoptera, Coleoptera and Diptera. In recent years, with the widespread use of diamide insecticides, many insects have developed a high level of resistance (Lai et al., 2011; Guo et al., 2014; Wang et al., 2018). Resistance mechanism studies suggested that the diamide insecticide resistance is associated with target mutation site of the ryanodine receptor (Troczka et al., 2015; Nauen and Steinbach, 2016). Field populations of *S*. *exigua* with a homozygous mutation in the ryanodine receptor gene I4743M have developed up to 154-fold higher levels of resistance to chlorantraniliprole (Zuo et al., 2019). Teng et al. (2022) found that the key mutation was I4743M in the resistant population of *S*. *exigua* in six regions of eastern China by sequencing the transmembrane region of RyR, and the mutation frequency was determined to be as high as 70%–100%. In this study, two potential mutation loci (G4911 and I4754) of the ryanodine receptor were examined in individuals from field populations of *G*. *daurica*, there were 12% of the heterozygous mutants G4911E in the population, while 32% of the heterozygous and 2% of heterozygous mutants were I4754M. The study showed that the G4946E and I4790M/K mutations in RyR were detected in resistant populations of *P*. *xylostella* (Guo et al., 2014; Troczka et al., 2015; Jouraku et al., 2020). It was confirmed that the G4946E and I4790M/K mutations could affect the binding of diamide insecticides to RyR by *in vitro* expression in Sf9 cells and construction of UAS-PxRyR transgenic *Drosophila* strain (Jouraku et al., 2020; Jiang et al., 2021; Richardson et al., 2021). Resistance to diamide insecticides in *S*. *frugiperda* is associated with mutations in the I4743M and G4891E loci of RyR (Boaventura et al., 2019). Therefore, the two amino acid sites G4911 and I4754 in *GdRyR* may be potential target binding sites for diamide insecticides, and mutations at the two sites could also be a potential resistance mechanism to diamide insecticides. In order to verify the inference, we analyzed the binding pattern of diamide insecticides to *GdRyR* and its mutants through protein homology modeling and molecular docking techniques.

The binding pattern analysis of insect RyR with diamide insecticides has been a hot topic of research in structural biology. Steinbach et al. (2015) had constructed a homology model of PxRyR, on which the G4946E and I4790M mutations were introduced, and the binding mode of diamide insecticides to PxRyR were analyzed, suggesting that the binding site of RyR to diamide insecticides may be close to the two mutation sites, but the exact binding mode, interaction force and residues forming the force are not clear. Studies on the mechanisms of resistance to diamide insecticides in Coleoptera are poorly reported. Based on the previous study, the rabbit ryanodine receptor (PDB: 7CF9) was selected as the template for constructing a homologous 3D model of *GdRyR*. In addition, *GdRyR*-E49111 and *GdRyR*-M4754 mutant models were constructed based on potential resistance allelic frequency detection by introducing the G4911E and I4754M mutations, and analyzed for their binding patterns to diamide insecticides. The results showed that mutating Gly of *GdRyR* to Glu resulted in reduced affinity to diamide insecticides. The current study showed that both of the G4946E mutation in *PxRyR* and the G4900E mutation in *SeRyR* can lead to resistance to diamide insecticides. Therefore, a substitution from Gly to Glu at 4911 position of *GdRyR* may lead to resistance to diamide insecticides on *G*. *daurica*. The frequency of mutations in *GdRyR* at G4911E should be a key concern for resistance risk assessment and reasonable applications of diamide insecticides for *G*. *daurica* control in future. The I4790M mutation of PxRyR and I4734M of SfRyR are also involved in insect resistance to diamide insecticides (Guo et al., 2014; Boaventura et al., 2019). The amino acid at position I4754 of *GdRyR* was not involved in its binding to diamide insecticides, and the substitution from Ile to Met may not reduce the affinity of *GdRyR* for diamide insecticides. Our result is similar with that of Teng et al. (2022), who reported that no significant correlation was found between chlorantraniliprole resistance level and RyR I4743M allele frequency in the six field populations of *S*. *exigua*. Therefore, the I4754M mutation may not be involved in the resistance to diamide insecticides on *G. daurica*, but whether the I4754M mutation can lead to resistance needs to be further explored in*in vitro* functional experiments.

## Data Availability

The datasets presented in this study can be found in online repositories. The names of the repository/repositories and accession number(s) can be found below: https://www.ncbi.nlm.nih.gov/genbank/, OP828593. http://www.wwpdb.org/, PDB: 7CF9.
